# Cause-Specific Mortality Due to Malignant and Non-Malignant Disease in Korean Foundry Workers

**DOI:** 10.1371/journal.pone.0088264

**Published:** 2014-02-05

**Authors:** Jin-Ha Yoon, Yeon-Soon Ahn

**Affiliations:** 1 Institute for Occupational Health, Yonsei University College of Medicine, Seoul, Korea; 2 Department of Preventive Medicine, Yonsei University Wonju College of Medicine, Wonju, Korea; 3 Department of Occupational and Environmental Medicine, Dongguk University Ilsan Hospital, Goyang, Korea; Kagoshima University Graduate School of Medical and Dental Sciences, Japan

## Abstract

**Background:**

Foundry work is associated with serious occupational hazards. Although several studies have investigated the health risks associated with foundry work, the results of these studies have been inconsistent with the exception of an increased lung cancer risk. The current study evaluated the mortality of Korean foundry workers due to malignant and non-malignant diseases.

**Methods:**

This study is part of an ongoing investigation of Korean foundry workers. To date, we have observed more than 150,000 person-years in male foundry production workers. In the current study, we stratified mortality ratios by the following job categories: melting-pouring, molding-coremaking, fettling, and uncategorized production work. We calculated standard mortality ratios (SMR) of foundry workers compare to general Korean men and relative risk (RR) of mortality of foundry production workers reference to non-production worker, respectively.

**Results:**

Korean foundry production workers had a significantly higher risk of mortality due to malignant disease, including stomach (RR: 3.96; 95% CI: 1.41–11.06) and lung cancer (RR: 2.08; 95% CI: 1.01–4.30), compared with non-production workers. High mortality ratios were also observed for non-malignant diseases, including diseases of the circulatory (RR: 1.92; 95% CI: 1.18–3.14), respiratory (RR: 1.71; 95% CI: 1.52–21.42 for uncategorized production worker), and digestive (RR: 2.27; 95% CI: 1.22–4.24) systems, as well as for injuries (RR: 2.36; 95% CI: 1.52–3.66) including suicide (RR: 3.64; 95% CI: 1.32–10.01).

**Conclusion:**

This study suggests that foundry production work significantly increases the risk of mortality due to some kinds of malignant and non-malignant diseases compared with non-production work.

## Introduction

Foundry work is an important component of the steel manufacturing industry that involves various processes in the casting of molten metal. During these processes, foundry workers can be exposed to toxic metals (e.g., chromium, manganese, lead, and cadmium), polycyclic aromatic hydrocarbons (PAH), crystalline silica, asbestos, benzene, formaldehyde, and carbon monoxide (CO) [1]. The extreme temperatures are most well-known characteristics of foundry process. The heavy physical workloads are occurred when pouring, moldings as well as fettling process are done by manually. These chemicophysical characteristic linked to various disease [2], and the heavy physical workload increased risk of fatal injury [3].

The risk of lung cancer among foundry workers is 1.5 to 2.5 times higher than the general population [4]. The high risk of lung cancer among foundry workers is an important health concern worldwide, and the International Agency for Research on Cancer considers foundry work as a significant cause of exposure to lung carcinogens [5]. Several studies have also reported that foundry workers have an increased risk of non-Hodgkin's lymphoma as well as nasopharyngeal, gastrointestinal, prostate, and kidney cancers [6]–[8]. However, the results of these studies have been inconsistent, and the risks of these cancers, excluding lung cancer, have not been extensively studied. Our large prospective cohort study therefore has the potential to contribute significantly to the current knowledge on the association between foundry work and cancer risk.

A few studies have also highlighted the risks of non-malignant diseases such as respiratory, autoimmune, and cardiovascular diseases [2] as well as fatal injuries among foundry workers [1], [3]. The level of exposure to crystalline silica among foundry workers often exceeds the acceptable level, and silica dust may disperse from the location where the castings and fettlings are done throughout the entire workplace [9]. Therefore, respiratory diseases, including silicosis and its related diseases, are a major health concern in foundries. Some epidemiologic evidence suggests that occupational exposures to crystalline silica may also be associated with renal and autoimmune diseases such as systemic sclerosis and rheumatoid arthritis [10]. In addition, Goodpasture's syndrome, the autoimmune disease of glomerulus, in foundry workers has been linked to a long-term exposure to crystalline silica [11]. Preventable deaths due to fatal injuries as well as suicide [1], [3] have also been implicated as a significant cause of death among foundry workers. Some researchers have suggested that the high risk of chronic disease and injury events are associated with a weak socioeconomic support system for foundry workers [12].

Studies that have analyzed the risk of non-malignant diseases among foundry workers are not frequently published, and the findings of the few published studies have been inconsistent. Critiques of these studies suggest a significant “healthy-worker effect” among foundry workers because the physically demanding nature of foundry work influences worker selection and survival [12], [13]. Large cohort studies that compare mortality ratios of foundry production workers with general population or non-production workers are needed to avoid this healthy-worker effect.

Our ongoing study has collected more than 150,000 person-years of data from Korean foundry production workers. Almost all Korean foundries were established after the 1960s, and are therefore relatively new. The mortality patterns of workers from more recently established foundries have not been well studied. Therefore, it is unknown whether the mortality pattern among Korean foundry workers is comparable to foundry workers from Western countries in which the foundries are generally older. Only three previous studies have investigated the health problems of Korean foundry workers. These studies indicated that the overall morbidity from cancer, including lung cancer, were higher in foundry workers compared with the general population. Furthermore, the prevalence of pneumoconiosis among Korean foundry workers was 3.7% [14]–[17]. The leading causes of death differ between Korean males and males from the Western population. Malignant diseases are the most common cause of death among the elderly Korean population, and lung, liver, and stomach cancer are the most common causes of death among all Koreans, with mortality rates of 31.7, 21.8, and 19.4 per 100,000 people, respectively [18]. The second most common cause of death below age 60 is suicide, and the second most common cause of death above age 60 is cardiovascular and cerebrovascular disease [18]. However, the mortality rates from malignant and non-malignant disease among Korean foundry worker have not been well studied. Our results evaluate the total mortality due to malignant and non-malignant diseases as well as to fatal injuries in a large cohort of Korean foundry workers. Job-specific mortality was stratified by the following job categories: melting-pouring, molding-coremaking, fettling, and uncategorized production work. In addition, the rate of suicide was studied independently from other injury-related deaths.

## Methods

### Ethics statement

This work was approved by the Institutional Review Board (IRB) of Dongguk University Ilsan Hospital (IRB SOP ver4.0_20100401:2010-1-48). All information used to write this paper was not hospital medical record. The cohort was constructed 10 years ago. This time, it is just followed the mortality through the mortality registry of Korea National Statistical Office (KNSO). So, IRB of Dongguk University was waived written consent of individual workers or their next of kin. In Korea the follow-up of the previous constructed cohort before Nov, 2012 is waived written consent, which is based on the premise that researchers protect personal information of all study subjects.

### Cohort definition

The Korean Iron Foundry (KIF) study is an ongoing multi-industrial cohort study that includes 17,098 workers at 208 iron and steel foundries who were working anytime between 1 January 1992 and 31 December 2000 [17]. The occupational history and the vital status were traced by retrospectively (from 1992 to 2000) as well as prospectively (after 2001). The follow-up period encompassed the first day of employment or 1 January 1992 (whichever date occurred later) until the date of death, 31 December 2007, or 31 December 2008 for cancer deaths only (whichever date occurred first). The death rate among female foundry workers, who usually hold non-production work, was relatively low; therefore, female workers were excluded from the data analysis. The cohort included in this current study was comprised of 14,611 male foundry workers.

Vital statistics have been linked with death records at the KNSO. This registry includes more than 99% of the death records of the general Korean population since 1992, when the cause of death information first became available. KNSO records provide the residence registration number (RRN; a unique 13-digit number assigned to all Koreans), cause of death (International Classification of Diseases, 10th Edition; ICD-10), and date of death for each record.

Reference mortality rates for the Korean male population were derived from the KNSO data for the years 1992 to 2007 (2008 for cancer mortality). The number of mortalities (i.e., the numerator of the mortality rate) are stratified by the type of disease (classified by the ICD-10), and age (in 5-year intervals). The total population (i.e., the denominator of the mortality rate) was obtained from a KNSO report of the registered population between the years 1992 and 2007 (2008 for cancer mortality) in which the population numbers were stratified by age (in 5-year intervals).

### Exposure assessment

The current study did not assess individual exposures. In this study, foundry work was classified as either production or non-production work, and production work was further classified into four categories: melting-pouring, molding-coremaking, fettling, and uncategorized production work. For workers who had more than two types of job categories, the job category with the longest duration was chosen for analysis. Workers who worked in more than two job categories with the same duration, these workers were classified as two job categories respectively. If there was not enough information to get defining job categories, although workers worked for production workers, these workers were classified as uncategorized.”. Only two job categories were used to calculate the Standardized Mortality Ratios (SMRs) to compare to the general Korean male population because 1) low rates of cancer across four job categories reduced statistical power; 2) some workers in the cohort had worked in more than 2 job categories at the same time; and 3) workers in different job categories at small companies do not work in separate locations, which suggests that workers have been exposed to similar hazards regardless of the job category [19]–[21]. Non-production workers were defined that who did not directly treat hazardous materials in their job. Hence, non-production workers' job included general affairs, human resources, purchase materials, accounting, secretary, driving, sales and other office work. However if some foundries were not well organized or separated their job systemically, the non-production workers somewhat exposed to the same hazards as production workers with relatively very low level. Also socioeconomic status, which affects the health, of non-production workers of foundry was similar to that of production workers. Because the scale of foundry was very small, the salary of non-production workers was not high comparing to that of production workers. Therefore non-production workers are chosen as relatively good reference to measure exposure effect of foundry production workers in this study.

### Statistical analysis

A classification table for the calculation of person-years and analysis of SMRs used the Person Year and Mortality Computation Program (PAMCOMP) [22]. Person-year observations were jointly classified into 5-year intervals, 3 calendar intervals (1992-1996, 1997–2002, 2003–2007), and 2 or 5 job categories (non-production or production, and non-production, melting-pouring, molding-coremaking, fettling, or uncategorized). Classification was based on a 5-year lag for all diseases. Hence, we didn't include the death within 5 years from the first employment. The SMRs and 95% confidence intervals (95% CI) were calculated in reference to the general Korean male population non-production worker. The relative risk (RR) of hazard ratios and 95% CI were calculated using by Cox-proportional hazard model for comparison of mortality ratios between production and non-production worker. “The relative risk (RR) of hazard ratios and 95% CI were calculated using by Cox-proportional hazard model for comparison of mortality ratios between production and non-production worker. The model includes such variables as job (production vs. non-production worker), age in 2001 (year of cohort construction), and duration of employment from the first employment to retirement (or quit to foundry work) (<10 years, 10≤ years) to control confounding variables. These analyses were performed using the Windows-based SPSS statistical package (version 17.0; SPSS, Chicago, IL). We analyzed the RRs in two or more cases of death in non-production workers to ensure statistical power.

## Results

### Demographics

In this study, we observed total of 187,734 person-years: 37,020 person-years for non-production workers and 150,714 person-years for production workers. Among the 150,714 person-years for production workers, 33,245 person-years were categorized into the melting-pouring group, 52,052 person-years were categorized into the molding-coremaking group, 37,343 person-years were categorized into the fettling group, and 37,096 person-years were categorized into the uncategorized group. Non-production workers comprised approximately 20% (37,020 person-years) of the cohort. The mean age at which individuals started foundry work was 25.4 years (32.8 years at their present foundry) and more than half (51.1%) of the foundry workers were first hired between the ages of 20 and 30 years old (Table 1).

**Table 1:** General character of foundry workers.

		Production workers		Non-production workers		Total workers	
		N	%	N	%	N	%
No. of workers		11,793	80.7	2,818	19.3	14,611	100.0
Person-years		150,714	80.3	37,020	19.7	187,734	100.0
Age (year) in 2001	20–29	1,533	13.0	620	22.0	2,153	14.7
	30–39	2,595	22.0	933	33.1	3,528	24.1
	40–49	3,856	32.7	696	24.7	4,552	31.2
	50–59	2,642	22.4	406	14.4	3,048	20.9
	60≤	1,167	9.9	163	5.8	1,330	9.1
	Mean age	44.7±11.3*		40.0±11.7*		43.8±11.0	
Year first employed at foundry	≤1979	5,213	44.2	1,131	40.1	6,344	43.4
	1980–1989	4,104	34.8	856	30.4	4,960	34.0
	≥1990	2,476	21.0	831	29.5	3,307	22.6
Age (year) first employed at foundry	< 20	2,854	24.2	437	15.5	3,291	22.5
	20 – ≤ 30	5,720	48.5	1,747	62.0	7,467	51.1
	30 – ≤ 40	2,382	20.2	493	17.5	2,875	19.7
	40 ≤	837	7.1	141	5.0	978	6.7
	Mean age	25.5±9.2		25.1±10.3		25.4±8.9	

* p<.01.

### SMRs of non-malignant diseases of foundry workers compared with the general Korean men

Across all foundry workers, 76 deaths occurred among 37,020 person-years of non-production workers and 585 deaths occurred among 150,714 person-years of production workers.

There were no significant increases in SMRs due to non-malignant disease among non-production workers (Table 2). The all-cause mortality from non-malignant disease was significantly lower in non-production workers compared with the general Korean men (SMR: 0.55; 95% CI: 0.43–0.69). The SMRs among non-production workers due to diseases of the circulatory system (SMR: 0.61; 95% CI: 0.39–0.92), digestive system (SMR: 0.54; 95% CI: 0.27–0.97) including liver diseases (SMR: 0.51; 95% CI: 0.23–0.97), fatal injury (SMR: 0.57; 95% CI: 0.36–0.85), and suicide (SMR: 0.41; 95% CI: 0.13–0.95) were significantly lower than the SMRs among the general Korean men.

**Table 2:** Standardized Mortality Ratio (SMR) of non-malignant disease (Reference: Korean men).

		Non-production	Production	Total
Person-years		37,020	150,714	187,734
All non-malignant death	N	76	585	661
	SMR	0.55	1.06	0.96
	95%CI	0.43–0.69	0.97–1.15	0.89–1.03
Disease of circulatory system	N	23	128	151
	SMR	0.61	0.89	0.84
	95%CI	0.39–0.92	0.75–1.06	0.71–0.98
Ischemic heart diseases	N	7	33	40
	SMR	0.8	0.92	0.9
	95%CI	0.32–1.65	0.64–1.30	0.64–1.23
Cerebrovascular disease	N	13	61	74
	SMR	0.65	0.83	0.79
	95%CI	0.34–1.11	0.63–1.06	0.62–0.99
Disease of respiratory system	N	3	25	28
	SMR	0.39	0.97	0.84
	95%CI	0.08–1.14	0.63–1.43	0.56–1.21
Pneumonia	N	2	8	10
	SMR	1.22	1.36	1.33
	95%CI	0.14–4.39	0.59–2.68	0.64–2.45
Disease of the digestive system	N	11	102	113
	SMR	0.54	1.13	1.03
	95%CI	0.27–0.97	0.93–1.38	0.85–1.23
Liver diseases	N	9	93	102
	SMR	0.51	1.16	1.04
	95%CI	0.23–0.97	0.94–1.42	0.85–1.27
Disease of the genitourinary system	N	3	9	12
	SMR	1.43	1.07	1.14
	95%CI	0.29–4.18	0.49–2.04	0.59–2.00
Injury, poisoning and external causes	N	23	219	242
	SMR	0.57	1.29	1.15
	95%CI	0.36–0.85	1.12–1.47	1.01–1.30
Suicide	N	5	61	66
	SMR	0.41	1.19	1.04
	95%CI	0.13–0.95	0.91–1.53	0.80–1.32

There were no significant differences in SMRs due to non-malignant disease among production workers compared with the general Korean men, except for fatal injury (Table 2). The SMR due to fatal injury among production workers (SMR: 1.29; 95% CI: 1.12–1.47) was significantly higher than the SMR in the general Korean men. The SMR due to pneumonia among foundry workers was 1.33, but this SMR was not significantly different from the general Korean men.

### SMRs of malignant diseases of foundry workers compared with the general Korean men

The all-cause mortality due to malignant disease was significantly lower among non-production workers compared with the general Korean men (SMR: 0.64; 95% CI: 0.46–0.87) (Table 3). In addition, the SMR of stomach cancer was significantly lower among non-production workers compared with the general Korean men (SMR: 0.33; 95% CI: 0.09–0.84). The mortality due to other malignant diseases, such as colon, liver, pancreatic, lung, bladder, and lymphohematopoietic cancer, was not significantly different in non-production workers compared with the general Korean men. There were also no significant differences in the SMRs due to malignant disease among production workers compared with the general Korean men.

**Table 3:** Standardized Mortality Ratio (SMR) of malignant disease (Reference: Korean men).

		Non-production	Production	Total
Person-years		38,017	154,584	192,600
All malignant diseases	N	41	274	315
	SMR	0.64	1.04	0.96
	95%CI	0.46–0.87	0.92–1.17	0.86–1.07
Stomach	N	4	53	57
	SMR	0.33	1.08	0.93
	95%CI	0.09–0.84	0.81–1.41	0.70–1.20
Colon	N	5	18	23
	SMR	1.21	1.06	1.09
	95%CI	0.39–2.83	0.63–1.68	0.69–1.64
Liver	N	14	73	87
	SMR	0.86	0.99	0.97
	95%CI	0.47–1.44	0.78–1.25	0.78–1.20
Pancreas	N	2	9	11
	SMR	0.74	0.78	0.78
	95%CI	0.08–2.66	0.36–1.49	0.39–1.39
Lung	n	9	56	65
	SMR	0.67	1.06	0.98
	95%CI	0.31–1.27	0.80–1.38	0.76–1.25
Urinary bladder	N	2	5	7
	SMR	2.91	2.04	2.23
	95%CI	0.33–10.51	0.66–4.77	0.90–4.60
Lympohematopoietic	N	2	16	18
	SMR	0.75	1.44	1.3
	95%CI	0.08–2.70	0.82–2.33	0.77–2.06

SMRs of malignant deaths according to employment duration [<10 years and 10≤ at the time of cohort construction (in 2001)] were analyzed. Overall cancer mortality of foundry production workers was not significantly decreased with duration of employment from an SMR = 1.06 at less than ten years to SMR = 1.03 at 10 or more years. However stomach and lung cancer mortality were not significantly increased with duration of employment from SMR = 0.93 and SMR = 0.99 at less than ten years to SMR = 1.32 and SMR = 1.14 at 10 or more years, respectively (data not shown).

### RRs due to non-malignant disease in production workers compared with non-production workers

There were 585 cases of non-malignant deaths among production workers, including 99 among melting-pouring workers, 204 among molding-coremaking workers, 149 among fettling workers, and 154 among uncategorized workers (Table 4). The all-cause mortality due to non-malignant disease was significantly higher among production workers compared with non-production workers (RR: 2.00; 95% CI: 1.57–2.56 across all production workers; RR: 1.50; 95% CI: 1.11–2.04 for melting-pouring workers; RR: 1.91; 95% CI: 1.46–2.51 for molding-coremaking workers; RR: 1.92; 95% CI: 1.45–2.55 for fettling workers; and RR: 2.52; 95% CI: 1.90–2.34 for uncategorized workers).

**Table 4:** Relative risk (RR) of hazard ratio of non-malignant deaths (compared with non-production workers).

		Production	Melting & pouring	Molding & core making	Fettling	Uncategorized
Person-years		150,714	33,245	52,052	37,343	37,096
All non-malignant death	N	585	99	204	149	154
	RR^a^	2.00	1.50	1.91	1.92	2.52
	95% CI	1.57–2.56	1.11–2.04	1.46–2.51	1.45–2.55	1.90–2.34
Diseases of the circulatory system	N	128	24	39	31	40
	RR	1.92	1.64	1.60	1.71	2.75
	95%CI	1.18–3.14	0.86–3.11	0.91–2.85	0.95–3.07	1.57–4.80
Ischemic heart diseases	N	33	9	7	8	9
	RR	1.60	1.94	0.85	1.44	1.86
	95%CI	0.66–3.88	0.67–5.61	0.28–2.55	0.49–4.25	0.65–5.34
Cerebrovascular diseases	N	61	8	21	17	21
	RR	1.85	1.01	1.64	1.93	2.91
	95%CI	0.93–3.68	0.42–2.85	0.75–3.61	0.85–4.35	1.34–6.30
Diseases of the respiratory system	N	25	3	6	5	11
	RR	2.68	1.38	1.74	1.66	5.71
	95%CI	0.79–9.15	0.26–7.29	0.41–7.44	0.39–7.06	1.52–21.42
Pneumonia	N	**8**	1	3	1	3
	RR	**1.11**	0.57	1.10	0.46	1.84
	95%CI	**0.23–5.34**	0.05–6.39	0.18–6.69	0.04–5.12	0.30–11.37
Diseases of the digestive system	N	102	13	48	20	23
	RR	2.27	1.29	2.95	1.68	2.21
	95%CI	1.22–4.24	0.58–2.89	1.53–5.68	0.81–3.51	1.07–4.56
Liver diseases	N	93	11	45	19	20
	RR	2.55	1.35	3.39	1.96	2.38
	95%CI	1.28–5.06	0.56–3.25	1.66–6.95	0.89–4.34	1.08–5.25
Diseases of the genitourinary system	N	9	0	2	0	**7**
	RR	0.94	–	0.49	–	3.39
	95%CI	0.24–3.63	–	0.08–2.97	–	0.84–13.70
Injury, poisoning and other consequences of external causes	N	219	49	72	60	47
	RR	2.36	2.34	2.22	2.51	2.28
	95%CI	1.52–3.66	1.44–3.95	1.37–3.57	1.54–4.09	1.37–3.79
Suicide	N	61	15	18	18	13
	RR	3.64	4.05	3.07	4.11	3.73
	95%CI	1.32–10.01	1.34–12.21	1.04–9.08	1.39–12.16	1.20–11.57

a Cox-proportional hazard model: stratified on age and employment duration.

Production workers had significantly higher mortality rates than non-production workers. These mortality rates were associated with the following: diseases of the circulatory system (RR: 1.92; 95% CI: 1.18–3.14) including cerebrovascular disease (RR: 1.48, 95%CI: 1.13–1.90) but not ischemic heart disease, diseases of the respiratory system (RR: 5.71; 95% CI: 1.52–21.42 for uncategorized production worker), diseases of the digestive system (RR: 2.27; 95% CI: 1.22–4.24) including liver disease (RR: 2.55; 95% CI: 1.28–5.06), and injury, poisoning, and other external causes (RR: 2.36; 95% CI: 1.52–3.66) including suicide (RR: 3.64; 95% CI: 1.32–10.01). Eight deaths were due to pneumonia and 9 deaths were due to diseases of the genitourinary system, and these deaths were not significantly related to production work.

When subgroup analyses were undertaken, the RR due to digestive systems including liver diseases were significantly increased in molding-coremaking and uncategorized workers (RR: 2.95; 95% CI: 1.53–2.89, RR: 2.21; 95% CI: 1.07–4.56 for digestive systems, RR: 3.39; 94% CI: 1.66–6.95, RR: 2.38; 95% CI:1.08–5.25 for liver disease, respectively). The mortality rate due to injury, poisoning, and other external causes, including suicide, was significantly higher among all subgroup compared with non-production worker.

### RRs due to malignant disease in production workers compared with non-production workers

There were 274 deaths due to malignant disease, including 55 among melting-pouring workers, 92 among molding-coremaking workers, 76 among fettling workers, and 65 among uncategorized workers (Table 5). The all-cause mortality due to malignant disease was significantly higher in production workers compared with non-production workers (RR: 1.90; 95% CI: 1.36–2.64 across all production workers, RR: 1.67; 95% CI: 1.11–2.53 among melting-pouring workers, RR: 1.66; 95% CI: 1.14–2.41 among molding-coremaking workers, RR: 1.85; 95% CI: 1.26–2.72 among fettling workers, and RR: 2.01; 95% CI: 1.35–2.99 among uncategorized workers).

**Table 5:** Relative risk (RR) of hazard ratio of malignant deaths (compared with non-production workers).

		Production	Melting & pouring	Molding & core making	Fettling	Uncategorized
Person-years		154,584	34,082	53,390	38,220	38,125
All malignant death	N	274	55	92	76	65
	RR^a^	1.90	1.67	1.66	1.85	2.01
	95%CI	1.36–2.64	1.11–2.53	1.14–2.41	1.26–2.72	1.35–2.99
Stomach	N	53	10	14	15	14
	RR	3.96	2.83	2.59	3.57	4.42
	95%CI	1.41–11.06	0.89–9.06	0.84–7.99	1.18–10.80	1.43–13.65
Colon	N	18	4	6	5	6
	RR	0.99	0.89	0.95	0.96	1.41
	95%CI	0.36–2.70	0.24–3.35	0.28–3.22	0.28–3.37	0.44–4.69
Liver	N	73	14	28	18	16
	RR	1.35	1.31	1.36	1.23	1.31
	95%CI	0.76–2.40	0.62–2.75	0.71–2.60	0.61–2.48	0.61–2.48
Pancreas	N	**9**	2	5	3	1
	RR	**1.25**	1.17	1.91	1.69	0.57
	95%CI	**0.27–5.93**	0.16–8.44	0.36–10.12	0.27–10.65	0.05–6.50
Lung	N	56	11	17	20	11
	RR	2.08	1.69	1.52	2.44	1.75
	95%CI	1.01–4.30	0.67–4.24	0.66–3.50	1.08–5.49	0.70–4.34
Urinary bladder	N	5	1	3	0	1
	RR	0.99	1.05	1.73	–	0.94
	95%CI	0.17–5.84	0.07–14.83	0.23–12.83	–	0.07–12.07
Lympohematopoietic	N	16	2	4	4	6
	RR	1.91	1.07	1.37	1.82	3.15
	95%CI	0.44–8.29	0.15–7.65	0.25–7.51	0.33–9.97	0.63–15.69

a Cox-proportional hazard model: stratified on age and employment duration.

The mortality rates were significantly higher among production workers compared with non-production workers for the following disease categories: stomach cancer (SMR: 3.96; 95% CI: 1.41–11.06), and lung cancer (RR: 2.08; 95% CI: 1.01–4.30). There were no significant differences in the mortality rates for colon, liver, pancreatic, bladder, or lympohematopoietic cancer.

In the subgroup analyses of melting-pouring and molding-coremaking workers the increased mortality due to malignant diseases was not significant. The fettling workers had higher mortality ratio in stomach and lung cancer compared with non-production workers (RR: 3.57; 95% CI: 1.18–10.80, RR: 2.44; 95% CI: 1.08–5.49, respectively). There were no significant increments of mortality in subgroup analyses of uncategorized workers.

The RRs were calculated according to employment durations between 10 or more year and less than 10 years. The RRs of overall cancer (RR = 1.14, 95%CI = 0.91–1.44) and lung cancer (RR = 1.07, 95%CI = 0.65–1.76) mortalities were not significantly increased at employment duration with 10 and more years comparing to at less than 10 years. However RR of stomach cancer mortality was significantly increased (RR = 1.83, 95%CI = 1.06–3.16) at 10 and more years (Data not shown).

## Discussion

This large retrospective and prospective cohort study found that foundry production workers have an increased risk of mortality due to both malignant and non-malignant diseases compared with foundry non-production workers. We stratified the production job categories into melting-pouring, molding-coremaking, fettling, and uncategorized production work. The cause-specific mortality rates among all production workers as well as within each job category are high enough to cause significant concern of occupational medicine.

Although the smoking status of an individual is a significant risk factor for various chronic diseases, including pulmonary and cardiovascular diseases, we did not adjust the SMRs and RRs by smoking history due to a lack of information. Therefore, the relatively higher mortality of lung cancer among production workers may be due to confounding effect of smoking. However, our previous survey in 2001 that examined 1,000 foundry workers, the smoking history of foundry production workers was not higher than either the general Korean male men or foundry non-production workers [17]. Also, three studies for evaluating pulmonary function of Korean foundry workers surveyed the smoking status. One study showed that the smoking rate (64.1%) of production workers significantly lower than those of non-production workers (74.5%) and Korean men (68.2%) [23]. Another two studies showed that the smoking rate of production workers was not significantly different from those of non-foundry workers [24], [25]. Furthermore, ischemic heart disease, the well-known smoking associated disease, is not increased among production workers in current study. Hence, these results suggest that the high mortality rates in production workers of current study might not be occupied by smoking history only. To elucidate the health effect of foundry work, further researches including personal smoking history are needed.

This current study shows that the all-cause mortality rate of foundry workers was lower than that of the general Korean men. However, there may be a significant healthy-worker effect among foundry workers because foundry work is physically demanding, which may influence worker selection and survival [12], [13]. The scale of Korean foundry was very small. The foundries which have more than one hundred workers occupied less than 10 percent among 208 foundries of this cohort. So, working environment is poor and work load is too heavy to endure foundry works. In Korea, foundry work is perceived to be “3D” (difficulty, dirty and dangerous) [17]. Therefore just healthy workers can endure it. This might cause severe healthy worker effect in Korean foundry. This healthy-worker effect is strong enough that even the lung cancer mortality of foundry workers was not as high as the general Korean men in the current study (table 2, 3), despite that foundry workers are at a significantly increased risk of exposure to lung carcinogens. Even though lung cancer mortality was not significantly increased with duration of employment from SMR = 0.99 at less than ten years to SMR = 1.14 at 10 or more years. The healthy-worker effect was observed in several diseases in which the risk of mortality in foundry workers was lower than the risk in the general population. The relatively short-term follow-up period in the current study may not be long enough to overcome the healthy-worker effect. To further control for the healthy-worker effect, our study included foundry non-production workers as an internal reference group. The results of the current study indicated that certain cause-specific mortality rates were higher among production workers compared with non-production workers even though these rates were not higher than the general Korean men. However, we could not confirm whether these low mortalities are due to health work effect or just means unrelatedness. Therefore, our results suggest that mortality rates among foundry workers are high enough compare to non-production workers, and warrant concern of occupational medicine.

Our study provides evidence to support an increased risk of lung cancer among total foundry production workers (table 5). Production workers are exposed to various lung carcinogens, such as crystalline silica, asbestos, chromium, cadmium, nickel, and PAHs. A study from Sweden reported that the exposure levels of dust and quartz in fettler and furnace workers were higher than that in other production workers [26]. Melting-pouring workers may be exposed to fumes from molten metal, whereas fettlers and molding-coremaking workers may be exposed to metallic dust and fumes that are produced during cutting, welding, and grinding [27]. Production workers are also exposed to PAHs that are formed and vaporized from pyrolysis products at the mold-metal interface. In our study, total production workers (RR: 2.08), especially fettling workers (RR: 2.44), had a high risk of mortality due to lung cancer.

Although the stomach cancer mortality of foundry workers was not higher than that of the general Korean men, our study showed that mortality due to stomach cancer was higher in production workers compared with non-production workers (RR: 3.96). The job-stratified analyses also showed high mortality risks of stomach cancer among fettling (RR: 3.57) and uncategorized job categories (RR: 3.57). Also RRs of stomach cancer mortality was significantly increased (RR = 1.83, 95%CI = 1.06–3.16) at 10 and more years comparing to at less than 10 years. These findings are supported by the results of another cohort study from the United Kingdom that followed more than 10,000 workers and found a significant increase in stomach cancer among production workers [28]. Furthermore, a nested case-control study from China [29] reported that production workers had an increased risk of stomach cancer even after adjusting for non-occupational risk factors such as smoking and dietary patterns. Another study has shown a considerable association between the pathogenesis of stomach cancer and exposure to N-nitrosamines in production workers [30]. N-nitrosamine compounds are well-known carcinogens, and the incidence of gastric cancer was increased when workers in the metal [31] and leather tanning [32] industries were exposed to high levels of N-nitrosamines. In foundry work, N-nitrosamines may be produced during molding-coremaking and casting operations [33]. A case-control study that investigated the association between gastric cancer and occupational exposures found an increased risk of gastric cancer among workers who were exposed to lead and crystalline silica [34]. Inhaled dust that is removed from the respiratory tract may be swallowed, and this dust may become an irritant in the gastric environment [35]. Furthermore fettling workers are exposed to metalworking fluids when cleaning and finishing were doing for foundry products [36]. Exposure history of metalworking fluids were associated to increased risk of stomach cancer in foundry workers [36]. In nested case-control study, the mortality of stomach cancer in automobile machinists was related with exposure history of metalworking fluid [37].

The ingredients in the organic resin binder for molds may be released into the workplaces environment through pyrolysis. Therefore, production workers may be exposed to several carcinogens that have been implicated in lymphohematopoietic cancer, such as formaldehyde, benzene, and PAHs [38]. A previous study from Korea reported that the level of formaldehyde in foundries was 0.12–0.06 ppm [17], and the highest exposure levels of benzene were 0.35–2.46 ppm [39]. The 3 most common sand resins are novolac, furan, and phenolicurethane (phenol-formaldehyde based), and the major products of thermal decompositions are phenol, furan, and benzene, respectively [40]. A study that aimed to identify the sources of benzene during the casting process suspected xylene-sulfonic acid, which is a solidifying agent, to be the source of benzene through thermal decomposition above 400°C [41], and found the mean concentration of the produced benzene to be 2.91 ppm (range 1.98–3.72 ppm). Therefore, production workers may be exposed to benzene even when benzene is not used as a raw material. Although there were no significant increments of RR among production workers, in the present study, 16 cases of lymphohematopoietic cancer were reported, and the risk of this cancer was big among production workers who were potentially exposed to these toxins compared with only 2 cases among non-production workers (RR: 1.91; 95% CI:0.44–8.29).

A recent meta-analysis from 40 systematically extracted results showed a positive but weak association between bladder cancer mortality and foundry work [8]. Naphthalamines, which have been associated with bladder cancer, may be generated from the heating of sand additives in foundry workplace [42]. In the current study, there were only 5 deaths from bladder cancer, and these deaths were not shown to be related to production work. Because some malignant diseases, including bladder cancer, have a low mortality rate and a high survival rate, it is difficult to detect an association between occupational exposures and these malignant diseases. Therefore, more follow-up years or an additional morbidity study is needed to determine the relationship between bladder cancer and foundry work.

Although the liver is the most common target organ for many toxic chemicals that individuals are exposed to within the work environment, there is a lack of evidence for a relationship between production work and mortality due to liver disease. Furthermore, in the current study, mortality from liver disease was not higher in production workers compared with the general Korean men. This result may be due to a strong healthy-worker effect. However, a well-designed cohort study from Germany [43] showed that production workers had an increased mortality due to liver disease that was most likely related to exposures to nitrosamines, PAH, and various solvents that were used in the foundry processes. Nitrosamines can be produced from dimethyl-ethylamine through molding-coremaking and casting operations, and it can also be contaminated with secondary and tertiary amines [33]. In the current study, more than 90% of the 120 deaths due to digestive system complications were related to liver disease. We also found an increased risk of non-malignant liver diseases in production workers compared with non-production workers. Our previous study showed that non-specific hepatic enzymes are higher in foundry production workers compared with the general Korean population [17], and that these high levels may be related to heavy alcohol use among production workers. Therefore, an analysis of the mortality due to liver disease in foundry production workers should control for alcohol consumption to determine the contribution of occupational exposures.

Foundry work requires extreme physical exertion with high aerobic demands and the lifting of static loads. The physical demands are exacerbated by shift-work schedules and job strain [44]. Furthermore, production workers are exposed to both extreme temperatures and carbon monoxide (CO) [5], which increase the risk of cardiac disease [45]. Exposure to CO may induce tissue hypoxia and worsen cardiovascular demand, which may potentially contribute to coronary artery disease in production workers. CO toxicity combined with heavy physical demands may contribute a synergic effect on cerebrovascular disease in production workers. Although this hypothesis is biologically plausible [44], [46], there has been no consistent consensus on toxic cardiac effects resulting from a moderate chronic exposure to CO. The geometric mean values of CO levels were 0.58–7.34 ppm in 30 foundries during 2001–2002 [47]; therefore, it is not clear whether these relatively lower exposures of CO are independently linked to mortality due to cardiovascular disease. In the present study, we observed an increased risk of cardiovascular disease among foundry workers (RR: 2.91; 95% CI: 1.34–6.30 in uncategorized worker), but the association between ischemic heart disease and production work could not be confirmed. The mortality study could not determine the association between the incidence of chronic disease and exposure to various occupational risk factors. Therefore, further morbidity studies are needed to elucidate the nature of the relationship between ischemic heart disease and production workers.

High levels of toxic gases, fumes, and mineral dust, including crystalline silica, are generated from various foundry work processes, such as melting, pouring, molding, and fettling. The high exposure levels of dust in foundry workers have been associated with various respiratory symptoms, including coughing, phlegm production, and wheezing, as well as a reduction in pulmonary function [48]. Furthermore, various respiratory abnormalities have been observed in production workers, and the results of a pulmonary function test were found to be inversely correlated with the duration of work after adjusting for age, height, and smoking status [49]. In addition, molding and furnace workers [49] have a 4- to 8-fold greater risk of developing pneumoconiosis compared with non-production workers [49]. In a cross-sectional study of 30 foundries in Korea [47], pneumoconiosis was diagnosed in 5.8% of a total of 872 workers. Exposure to crystalline silica increases the risk of various respiratory abnormalities, including pulmonary tuberculosis and chronic obstructive pulmonary disease (COPD). Although the mortality across all production foundry workers was not higher than the general Korean men due to the healthy-worker effect, the results of our current study show that mortality due to respiratory diseases was significantly increased in uncategorized production workers compared with non-production workers in table 4 (RR: 5.71; 95% CI:1.52–21.42).

Injuries occur in relatively younger workers due to work inexperience. Injuries can result in a severe loss of potential working years compared with chronic disease and cancer [50]. Unintentional injuries are preventable, and investigations into the causes of occupational injuries are critical to the improvement of occupational health in foundry production workers. Production workers have a high risk of injury due to exposure to molten metal, heat, dust, fumes, gases and vapors, noise, vibration, strenuous physical activity, and heavy machinery [51]. The healthy-worker effect does not relate to accidental injuries. Therefore, the production workers in our study had a higher risk of injuries compared with both non-production workers and the general Korean men (table 2, 4).

Almost 30% of deaths as a result of injury were linked to suicide. Several studies have reported different suicide rates across various occupations. These studies have reported an elevated risk for suicide among workers with low incomes, high stress levels, and/or easy access to lethal means [52]. Nevertheless, a study from New Zealand showed that easy access to lethal means could not fully explain the high suicide risk among certain occupations [53]. Suicide is linked to psychiatric illness [54], but the risk of suicide may also be influenced by occupational stress as well as a low socioeconomic support system. The production workers in our study had a high risk of mortality due to suicide. This finding raises social concerns that may promote further investigations to elucidate the reasons for suicide among vulnerable occupations.

10 years cut-point of employment duration used to examine the possibility that longer employment duration in production work affect the higher mortality of stomach cancer (significant increase) or lung cancer risk (non-significant increase). In this study, employment duration was measured at the time of cohort construction (from the date of first employment to 31 December 2000). The duration of employment from 1 Jan 2001 to 31 Dec 2008(end of follow-up) was not reflected to calculate it, which might be cause unclear exposure effect to lung cancer mortality.

Our study, which showed an increased risk of mortality due to both malignant and non-malignant disease in male production workers compared with foundry non-production workers, had some limitations. In general, chronic disease reflects an individual's lifestyle, including smoking history, alcohol use, and obesity. However, our current study did not incorporate these lifestyle factors as confounders. Furthermore, malignant disease specific contributing factors were not taken into account when considering various cancers of death in this analysis, either. These lacks of information about individual or diseases specific risk factors are main limitation to elucidate the health effect of foundry work. A disease-specific study design that incorporates confounding factors is necessary to elucidate the association between foundry work and various diseases. we cannot know the incident date of injury because this data is mortality data, not incident data. The mortality was regarded as post retire event when worker died after retire; even the injury occurred prior to retire. Although that severe difference of injury mortality between production and non-production workers was seems to be related to unsafe workplaces of foundry process, our current study has limitation to explain that. Further analyses were needed to ensure the retirement effect to various diseases such as suicide and fatal injury.

In summary, foundry production workers had a high risk of mortality due to non-malignant diseases of the circulatory, respiratory, and digestive systems, as well as injury and malignant diseases such as stomach, lung cancers, compared with foundry non-production workers. In addition, we suggest that socioeconomic factors may affect the mortality rates of foundry workers. Therefore, multilateral approaches are needed to prevent mortalities related to foundry work. Future morbidity studies are needed to elucidate the association between various diseases and the occupational environment of foundry workers.

## References

[1] Park RM, Ahn YS, Stayner LT, Kang SK, Jang JK (2005) Mortality of iron and steel workers in Korea. Am J Ind Med 48: 194–204. 16094610 10.1002/ajim.20197

[2] Koskela RS, Mutanen P, Sorsa JA, Klockars M (2000) Factors predictive of ischemic heart disease mortality in foundry workers exposed to carbon monoxide. American journal of epidemiology 152: 628–632. 11032157 10.1093/aje/152.7.628

[3] Barreto SM, Swerdlow AJ, Smith PG, Higgins CD, Andrade A (1996) Mortality from injuries and other causes in a cohort of 21,800 Brazilian steel workers. Occupational and environmental medicine 53: 343–350. 8673183 10.1136/oem.53.5.343 PMC1128478

[4] Sherson D, Svane O, Lynge E (1991) Cancer incidence among foundry workers in Denmark. Arch Environ Health 46: 75–81. 2006897 10.1080/00039896.1991.9937432

[5] IARC (1987) Overall evaluatios of carcinogenicity: An updating of IARC monographs volumes 1–42, supplement 7. Lyon, France. International Agency for Research on Cancer: 224–225.

[6] Scott CS, Jinot J (2011) Trichloroethylene and cancer: systematic and quantitative review of epidemiologic evidence for identifying hazards. Int J Environ Res Public Health 8: 4238–4272. 22163205 10.3390/ijerph8114238 PMC3228569

[7] Porru S, Placidi D, Carta A, Gelatti U, Ribero ML, et al (2001) Primary liver cancer and occupation in men: a case-control study in a high-incidence area in Northern Italy. Int J Cancer 94: 878–883. 11745492 10.1002/ijc.1538

[8] Gaertner RR, Theriault GP (2002) Risk of bladder cancer in foundry workers: a meta-analysis. Occup Environ Med 59: 655–663. 12356924 10.1136/oem.59.10.655 PMC1740225

[9] Andersson L, Bryngelsson IL, Ngo Y, Ohlson CG, Westberg H (2012) Exposure assessment and modeling of quartz in Swedish iron foundries for a nested case-control study on lung cancer. Journal of occupational and environmental hygiene 9: 110–119. 22239127 10.1080/15459624.2011.645397

[10] Parks CG, Conrad K, Cooper GS (1999) Occupational exposure to crystalline silica and autoimmune disease. Environmental health perspectives 107 Suppl 5793–802. 10970168 10.1289/ehp.99107s5793 PMC1566238

[11] Kim MG, Koh DH, Lee SW, Jo M, H, Yoo HY, et al (2008) A Case of Goodpasture's Syndrome in a Foundry Worker. Korean J Occup Environ Med 20: 46–53.

[12] Koskela RS, Jarvinen E, Korhonen H, Mutanen P (1983) Health selection among metal workers. Scandinavian journal of work, environment & health 9: 155–161. 10.5271/sjweh.2430 6648413

[13] Koskela RS (1997) Mortality, morbidity and health selection among metal workers. Scandinavian journal of work, environment & health 23 Suppl 21–80. 9314171

[14] Ahn YS, Kim KS, Chung HK, Whang IS, Roh J (1997) Pneumoconiosis with workers of manufacturing industry in Incheon. Korean Journal of Occupational and Environmental Medicine 9: 589–603.

[15] Ahn YS, Park RM, Stayner L, Kang SK, Jang JK (2006) Cancer morbidity in iron and steel workers in Korea. American journal of industrial medicine 49: 647–657. 16804912 10.1002/ajim.20337

[16] Ahn YS, Song JS, Kang SK, Chung HK (2000) Understanding the occurrence of lung cancer in foundry workers through health insurance data. Korean Journal of Preventive Medicine 33: 299–305.

[17] Ahn YS, Won JU, Park RM (2010) Cancer morbidity of foundry workers in Korea. J Korean Med Sci 25: 1733–1741. 21165287 10.3346/jkms.2010.25.12.1733 PMC2995226

[18] Korea National Statistical Office. The Cause of Death Statistic at 2011. Available: http://kostat.go.kr/portal/korea/kor_nw/2/6/2/index.board?bmode=read&aSeq=260046. Accessed on 25 Aug 2013.

[19] Phee Y, Roh Y, Lee K, Kim H, Kim Y, et al (1997) Analysis of quartz content and particle size distribution of airborne dust from selected foundry operations. Korean Ind Hyg Assoc J 7: 196–208.

[20] Kim H, Roh Y, Phee Y, Won J, Kim Y (1998) Analysis of quartz contents by XRD and FTIR in respirable dust from various manufacturing industries part I-Foundry. J Korean Soc Occup Environ Hyg 8: 50–66.

[21] Park Y, Roh Y, Kim H, Han J, Ahn Y, et al (2003) A study of respirable dust concentrations and quartz contents in foundry. J Korean Soc Occup Environ Hyg 13: 90–97.

[22] Taeger D, Sun Y, Keil U, Straif K (2000) A stand-alone windows applications for computing exact person-years, standardized mortality ratios and confidence intervals in epidemiological studies. Epidemiology 11: 607–608. 10955416 10.1097/00001648-200009000-00019

[23] Koo JW, Chung CK, Park CY, Lee S-H, Lee K-S, et al (2000) The effect of silica dust on ventilatory function of foundry workers. JOURNAL OF OCCUPATIONAL HEALTH-ENGLISH EDITION- 42: 251–257.

[24] Choi JK, Rhee CO, Paek DM, Choi BS, Shin YC, et al (1994) Respiratory Health of Foundry Workers Exposed to Binding Resin. Korean Journal of Preventive Medicine 27: 274–285.

[25] Kim SY, Kim JI, Jung JH, Choi SH, Jung KY (2011) Lung Function in Workers at Small Foundries. Korean Journal of Occupational and Environmental Medicine 23: 317–323.

[26] Andersson L, Bryngelsson IL, Ohlson CG, Naystrom P, Lilja BG, et al (2009) Quartz and dust exposure in Swedish iron foundries. J Occup Environ Hyg 6: 9–18. 18982534 10.1080/15459620802523943

[27] Horng CJ, Horng PH, Hsu JW, Tsai JL (2003) Simultaneous determination of urinary cadmium, cobalt, lead, and nickel concentrations in steel production workers by differential pulse stripping voltammetry. Arch Environ Health 58: 104–110. 12899211 10.3200/AEOH.58.2.104-110

[28] Sorahan T, Faux AM, Cooke MA (1994) Mortality among a cohort of United Kingdom steel foundry workers with special reference to cancers of the stomach and lung, 1946–90. Occup Environ Med 51: 316–322. 8199681 10.1136/oem.51.5.316 PMC1127976

[29] Xu Z, Brown LM, Pan GW, Liu TF, Gao GS, et al (1996) Cancer risks among iron and steel workers in Anshan, China, Part II: Case-control studies of lung and stomach cancer. Am J Ind Med 30: 7–15. 8837676 10.1002/(SICI)1097-0274(199607)30:1<7::AID-AJIM2>3.0.CO;2-#

[30] Raj A, Mayberry JF, Podas T (2003) Occupation and gastric cancer. Postgrad Med J 79: 252–258. 12782770 10.1136/pmj.79.931.252 PMC1742699

[31] Ekstrom AM, Eriksson M, Hansson LE, Lindgren A, Signorello LB, et al (1999) Occupational exposures and risk of gastric cancer in a population-based case-control study. Cancer Res 59: 5932–5937. 10606238

[32] Krstev S, Dosemeci M, Lissowska J, Chow WH, Zatonski W, et al (2005) Occupation and risk of stomach cancer in Poland. Occup Environ Med 62: 318–324. 15837853 10.1136/oem.2004.015883 PMC1741010

[33] Ducos P, Gaudin R, Maire C, Mavelle T, Bouchikhi B, et al (1988) Occupational exposure to volatile nitrosamines in foundries using the “Ashland” core-making process. Environ Res 47: 72–78. 3168966 10.1016/s0013-9351(88)80022-7

[34] Parent ME, Siemiatycki J, Fritschi L (1998) Occupational exposures and gastric cancer. Epidemiology 9: 48–55. 9430268

[35] Cocco P, Ward MH, Buiatti E (1996) Occupational risk factors for gastric cancer: an overview. Epidemiol Rev 18: 218–234. 9021314 10.1093/oxfordjournals.epirev.a017927

[36] Park RM (2001) Mortality at an automotive engine foundry and machining complex. J Occup Environ Med 43: 483–493. 11382184 10.1097/00043764-200105000-00009

[37] Sullivan P, Eisen E, Kreibel D, Woskie S, Wegman D (2000) A nested case-control study of stomach cancer mortality among automobile machinists exposed to metalworking fluid. Ann Epidemiol 10: 480–481. 10.1016/s1047-2797(00)00120-4 11018429

[38] Tossavainen A (1990) Estimated risk of lung cancer attributable to occupational exposures in iron and steel foundries. IARC Sci Publ: 363–367. 2228134

[39] Ahn YS (2007) Occupational malignant lymphohematopoietic diseases compensated under the industrial accident compensation insurance from 1996 to 2005. Korean Journal of Occupational and Environmental Medicine 19: 81–92.

[40] Dungan RS, Reeves JB 3rd (2005) Pyrolysis of foundry sand resins: a determination of organic products by mass spectrometry. J Environ Sci Health A Tox Hazard Subst Environ Eng 40: 1557–1567. 15991723 10.1081/ese-200060630

[41] Oh DS, Lee SM, Lee BJ, Kim YJ (2006) A study on the Identification of Sources for Benzene Detected in the Casting Process. Journal of Korean Society of Occupational and Environmental Hygiene 16: 27–35.

[42] Hansen ES (1991) Cancer mortality among Danish molders. Am J Ind Med 20: 401–409. 1928115 10.1002/ajim.4700200312

[43] Adzersen KH, Becker N, Steindorf K, Frentzel-Beyme R (2003) Cancer mortality in a cohort of male German iron foundry workers. Am J Ind Med 43: 295–305. 12594777 10.1002/ajim.10187

[44] Koskela RS, Mutanen P, Sorsa JA, Klockars M (2000) Factors predictive of ischemic heart disease mortality in foundry workers exposed to carbon monoxide. Am J Epidemiol 152: 628–632. 11032157 10.1093/aje/152.7.628

[45] Steenland K (1996) Epidemiology of occupation and coronary heart disease: research agenda. Am J Ind Med 30: 495–499. 8892556 10.1002/(SICI)1097-0274(199610)30:4<495::AID-AJIM16>3.0.CO;2-#

[46] Palda VA (2003) Is foundry work a risk for cardiovascular disease? A systematic review. Occup Med (Lond) 53: 179–190. 12724552 10.1093/occmed/kqg052

[47] Ahn YS (2005) Respiratory diseases in foundry workers(Training materials for occupational respiratory diseases). In: Agency KOSH, editor. Incheon pp. 171–190.

[48] Gomes J, Lloyd OL, Norman NJ, Pahwa P (2001) Dust exposure and impairment of lung function at a small iron foundry in a rapidly developing country. Occup Environ Med 58: 656–662. 11555687 10.1136/oem.58.10.656 PMC1740042

[49] Kuo HW, Chang CL, Liang WM, Chung BC (1999) Respiratory abnormalities among male foundry workers in central Taiwan. Occup Med (Lond) 49: 499–505. 10658302 10.1093/occmed/49.8.499

[50] Barreto SM, Swerdlow AJ, Smith PG, Higgins CD, Andrade A (1996) Mortality from injuries and other causes in a cohort of 21,800 Brazilian steel workers. Occup Environ Med 53: 343–350. 8673183 10.1136/oem.53.5.343 PMC1128478

[51] Barreto SM, Swerdlow AJ, Smith PG, Higgins CD (1997) A nested case-control study of fatal work related injuries among Brazilian steel workers. Occup Environ Med 54: 599–604. 9326164 10.1136/oem.54.8.599 PMC1128985

[52] Agerbo E, Gunnell D, Bonde JP, Mortensen PB, Nordentoft M (2007) Suicide and occupation: the impact of socio-economic, demographic and psychiatric differences. Psychol Med 37: 1131–1140. 17445281 10.1017/S0033291707000487

[53] Skegg K, Firth H, Gray A, Cox B (2010) Suicide by occupation: does access to means increase the risk? Aust N Z J Psychiatry 44: 429–434. 20397784 10.3109/00048670903487191

[54] Maris RW (2002) Suicide. Lancet 360: 319–326. 12147388 10.1016/S0140-6736(02)09556-9

